# Obinutuzumab for the treatment of podocytopathy with concurrent suspected but unconfirmed B-cell lymphoproliferative disorder: a case report and literature review

**DOI:** 10.3389/fimmu.2026.1803073

**Published:** 2026-04-13

**Authors:** Yong-Zhe Zhang, Rui Yu, Xiao-Mei Liu, Guo-qing Shi, Mi Tian, Hua Zhou, Bei-Ru Zhang

**Affiliations:** 1 Department of Nephrology, Shengjing Hospital of China Medical University, Shenyang, Liaoning, China; 2 Department of Emergency, The Fourth Affiliated Hospital of China Medical University, Shenyang, Liaoning, China

**Keywords:** acute kidney injury, B-cell lymphoma, nephrotic syndrome, obinutuzumab, podocytopathy, suspected but unconfirmed B-cell lymphoproliferative disorder

## Abstract

B-lymphocyte proliferative disorder have a certain incidence rate among the elderly. Whether these conditions require treatment is closely related to the presence of concurrent organ damage. This case report details the diagnostic journey and treatment process of a 76-year-old male patient who presented with nephrotic syndrome and acute kidney injury. His condition was further complicated by the presence of monoclonal M proteinemia and the malignant B-lymphocytes in the bone marrow. Through a series of examinations, a diagnosis of podocytopathy with suspected but unconfirmed B-cell lymphoproliferative disorder was established. Treatment involved with CD20 monoclonal antibody (Obinutuzumab). The patient achieved complete remission of proteinuria and normalization of renal function within one month, sustained at one-year follow-up. While the underlying mechanism remains uncertain, whether direct immunomodulation, B-cell clone control or both. This is the first reported case of successful treatment of podocytopathy with concurrent suspected but unconfirmed B-cell lymphoproliferative disorder using Obinutuzumab, offering a new therapeutic way for similar complex medical conditions.

## Introduction

Podocytopathy, including minimal change disease (MCD) and focal segmental glomerulosclerosis (FSGS), are common causes of nephrotic syndrome. These conditions are characterized by podocyte injury, leading to proteinuria, edema, and hypoalbuminemia. The coexistence of podocytopathies with B-lymphocyte proliferative disorder, presents diagnostic and therapeutic challenges. In the past reports, MCD is known to be associated with Hodgkin’s disease and other lymphomas, particularly in the setting of steroid-resistant or relapsing MCD, Hodgkin’s disease should be considered as a potential underlying risk factor and a detailed lymph node examination should be performed (1–3). There are also reported cases about non-Hodgkin’s lymphoma associated with glomerulonephritis (1, 4, 5). Although the pathogenetic relationship between lymphoma and podocytopathy is not fully understood, the observed association may attributed to circulating tumor-secreted cytokines or other leukocyte-derived factors that affect glomerular function (1). Specific recommendations regarding the management of glomerulopathies accompanied by suspected but unconfirmed B-cell lymphoproliferative disorder are lacking. Cases of patients with MCD or FSGS that not respond well to immunosuppressive therapy but get remission after chemotherapy for lymphoma imply a possibly pathophysiologic connection of these conditions (6).

This report details the diagnostic and therapeutic process of an elderly male patient who presented with intermittent bilateral lower extremity edema and reduced urine output. During routine examinations, monoclonal M protein was detected in his blood and urine. Subsequent bone marrow aspiration revealed suspected B-cell lymphocytes, and renal pathology demonstrated podocytopathy. Notably, the patient achieved renal remission following Obinutuzumab therapy, providing valuable insights for the diagnosis and management of similar cases. This case, characterized by puzzling clinical manifestations, a complex diagnostic journey, and a multidisciplinary treatment course, aims to explore more effective, personalized therapeutic strategies for podocytopathy with concurrent suspected but unconfirmed B-cell lymphoproliferative disorder.

## Case presentation

A 76-year-old male patient developed symptoms of bilateral lower extremity edema without an obvious cause for more than half a month and simultaneously perceived a decrease in urine output, with a 24-hour urine volume of approximately 600ml. The symptoms persisted for 3 days before the patient sought medical attention at an outpatient clinic. The laboratory tests conducted at that hospital indicated: urine protein 3+; serum albumin 24.7g/L; creatinine 64.9umol/L, he was given symptomatic treatment. Due to the edema progressively worsening over the past 15 days, the patient visited our hospital and the laboratory tests revealed: urine protein 4+; albumin 23.9g/L; creatinine 180umol/L(eGFR:30.8mL/min/1.73m^2^). The patient was then admitted to the nephrology ward of our hospital and preliminarily diagnosed with nephrotic syndrome, acute kidney injury.

Further detailed consultations and examinations were then conducted. The patient reported a history of good health, with no prior discomfort or significant medical issues including hypertension and diabetes. Family history was non-contributory, with no known hematologic or renal disorders. He was a non-smoker, did not consume alcohol, and had no recent medication use or travel history. Physical examination revealed the patient’s vital signs were stable (T: 37.0 °C, P: 66 beats/min, R: 16 breaths/min, BP: 121/81 mmHg, oxygen saturation 99% on room air). His skin was intact with no rashes, petechiae, or ecchymosis. There was no palpable lymphadenopathy in the cervical, axillary, supraclavicular, or inguinal regions. Head and neck examination was unremarkable, with no conjunctival pallor or scleral icterus. Chest examination revealed clear breath sounds bilaterally and regular heart sounds without murmurs. Abdomen was soft, non-tender, with no hepatosplenomegaly or palpable masses. Extremities showed severe, symmetric, pitting edema with intact peripheral pulses. Neurologic examination was normal. Laboratory findings confirmed nephrotic syndrome (24h protein 13.2 g/d, albumin 17.6 g/L, cholesterol 8.12 mmol/L) with acute kidney injury (creatinine 180 μmol/L). Immunofixation revealed IgM-κ monoclonal bands in serum and κ monoclonal band in the urine. Serum free light chain κ/λ ratio was 1.556 (reference range 0.31–1.56). Immunoglobulins showed suppressed IgG (<2.5 g/L) and IgM was elevated at 3.51 g/L (reference range 0.5–2.2 g/L). Autoimmune markers, including Cryoglobulin were unremarkable. Laboratory Examinations showed as **Table 1**. Imaging studies revealed no significant abnormalities. Renal ultrasound showed normal-sized kidneys. Echocardiography was normal, with no evidence of myocardial hypertrophy. Chest and abdominal CT scans revealed no evidence of lymphadenopathy, organomegaly, or mass lesions.

**Table 1:** Summary of major laboratory and imaging examination results.

Category	Parameter	Result	Reference range
Blood Routine	Hemoglobin (g/L)	123	130–175
	White Blood Cells (×10^9^/L)	4.1	3.5–9.5
	Platelets (×10^9^/L)	133	125–350
Urinalysis	Urine Protein (dipstick)	4+	Negative
	Red Blood Cells (/HPF)	21	0–3
	Dysmorphic RBCs (%)	70%	Negative
	24h Proteinuria (g/d)	13.2	<0.15
Blood Biochemistry	Total Protein (g/L)	38	65–85
	Globulin (g/L)	20.4	20–40
	Albumin (g/L)	17.6	35–53
	Creatinine (μmol/L)	180	57–111
	Potassium (mmol/L)	3.9	3.5–5.3
	Corrected Calcium (mmol/L)	2.1	2.11–2.52
	Total Cholesterol (mmol/L)	8.12	0–5.2
	Triglycerides (mmol/L)	1.57	0–1.7
	HDL-C (mmol/L)	2.02	>1.0
	LDL-C (mmol/L)	3.66	<3.4 (optimal)
Immunoglobulins	IgG (g/L)	<2.5	8–16
	IgA (g/L)	2.54	0.7–3.3
	IgM (g/L)	3.51	0.5–2.2
Serum Free Light Chains	κ FLC (mg/L)	196	6.7–22.4
	λ FLC (mg/L)	126	8.3–27.0
	κ/λ Ratio	1.556	0.31–1.56
Urine Free Light Chains	κ FLC (mg/L)	96.3	0–7.09
	λ FLC (mg/L)	59.1	0–4.27
	κ/λ Ratio	1.63	0.75–4.5
Immunofixation Electrophoresis	Serum	IgM-κ monoclonal band	Negative
	Urine	κ monoclonal band	Negative
Immunologic Profile	C3 (g/L)	1.69	0.8-1.6
	C4 (g/L)	0.39	0.2-0.4
	ANCA	Negative	Negative
	Anti-GBM Antibody	Negative	Negative
	ANA	Negative	Negative
	Cryoglobulin	Negative	Negative

HB, Hemoglobin; WBC, White Blood Cells; PLT, Platelets; Prot, Protein; RBC, Red Blood Cells; Abn. RBCs, Abnormal Red Blood Cells; 24h Prot., 24-hour Proteinuria; HPF, High-Power Field; TP, Total Protein; GLB, Globulin; ALB, Albumin; Cr, Creatinine; K^+^, Potassium; Ca²^+^ corr., Corrected Calcium; TC, Total Cholesterol; TG, Triglyceride; HDL-C, HDL Cholesterol; LDL-C, LDL Cholesterol; IgG, Immunoglobulin G; IgA, Immunoglobulin A; IgM, Immunoglobulin M; FLC-κ, Free Light Chain Kappa; FLC-λ, Free Light Chain Lambda; FLC-κ/λ Ratio, Free Light Chain Kappa/Lambda Ratio; uFLC-κ, Urinary Free Light Chain Kappa; uFLC-λ, Urinary Free Light Chain Lambda; uK/λ Ratio, Urinary Kappa/Lambda Ratio; Serum Prot., Serum Protein Band; Urine Prot., Urine Protein Band; κ-M Prot., Kappa-type M Protein; ANCA, Anti-Neutrophil Cytoplasmic Antibodies; Anti-GBM Ab, Anti-Glomerular Basement Membrane Antibody; ANA, Antinuclear Antibodies; C3, Complement Component 3; C4, Complement Component 4.

1. Renal biopsy revealed no evidence of monoclonal immunoglobulin deposition by immunofluorescence or electron microscopy.

2. All reference ranges have been standardized according to institutional laboratory norms.

Bone marrow aspiration showed hyperplasia with 0.8% plasma cells. Flow cytometry revealed 6.91% malignant monoclonal B lymphocytes (expressing CD45, CD20, CD19, CD22, CD79b, CD81, cBcl-2, cKappa; partial CD23/CD200; negative for CD10, CD5, CD25, FMC7, sIgM, CD43, CD103, CD11c, CD38, CD138, Ki-67, or cLambda). Cells were small malignant monoclonal mature B lymphocytes. CD3+ T cells (13.95%) and CD4+/CD8+ ratio (0.64) were normal. CD56+ NK cells comprised 3.42% of the total. It was suggestive of B-cell lymphoma.

**Figure 1:**
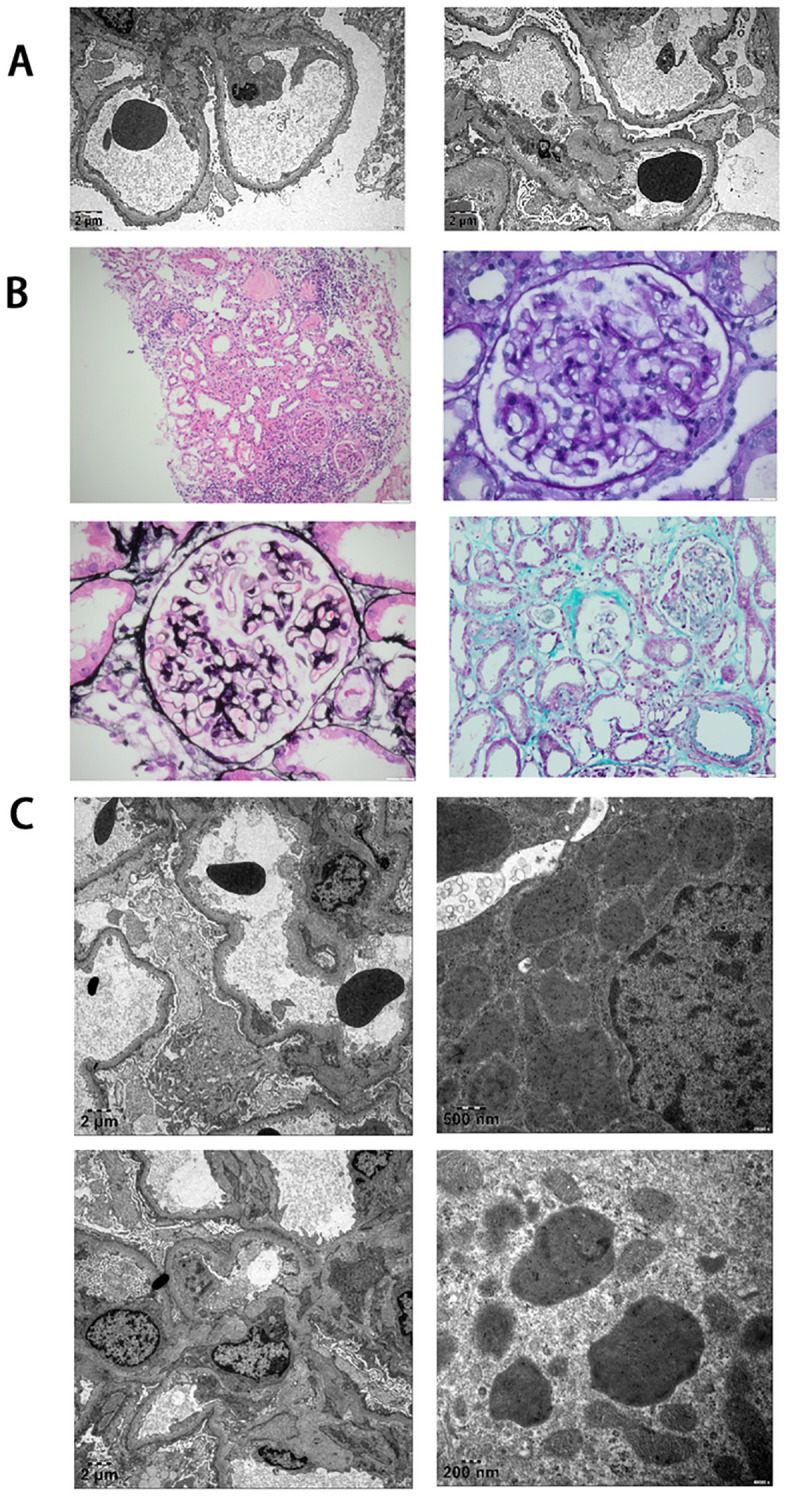
Renal pathological changes in the patient. **(A)** Electron microscopic findings on renal biopsy. Extensive effacement of foot processes is evident. **(B)** Light microscopic findings on renal biopsy. Minor glomerular abnormalities are observed. Staining techniques and magnifications: HE (Hematoxylin and Eosin) at ×100; PAS (Periodic Acid-Schiff) at ×400; PASM (Periodic Acid-Silver Methenamine) at ×400; Masson at ×400. **(C)** Immunoelectron microscopic findings on renal biopsy. No restricted deposition of Kappa and Lambda light chains is detected.

Renal biopsy was performed to identify kidney disease. Electron microscopy of renal tissue showed diffuse fusion of glomerular foot processes, with a small quantity electron-dense deposits in the mesangial region, consistent with podocyopathy (**Figure 1A**). The renal tissue’s pathological features under light microscopy, as demonstrated by Hematoxylin and Eosin (HE), Periodic Acid-Schiff (PAS), Periodic Acid-Silver Methenamine (PASM) and Masson staining revealed 24 glomeruli with 7 (29.2%) exhibiting global sclerosis without segmental involvement, while the remaining glomeruli displayed mild pathological changes. Focal tubular atrophy was observed, affecting approximately 25% of the area with concentrated distribution, accompanied by renal interstitial edema. Periglomerular infiltration of lymphocytes, monocytes, and eosinophils was observed surrounding sclerotic glomeruli. No lymphocytes with abnormal morphology were observed (**Figure 1B**). Immunofluorescence analysis, conducted under frozen section microscopy, observed 5 glomeruli without detection of glomerular sclerosis. The results were as follows: IgG (+/-); IgG1 (+/-); IgG2 (-); IgG3 (-); IgG4 (-); IgA (-); C3 (-); C1q (-); IgM (+); Fib (-); Alb (-); Kappa (+/-); Lambda (+/-); PLA2R (-).The manifestations observed under light microscopy further confirmed that the patient’s renal pathology was consistent with podocytopathy. No significant immune complex deposition was observed. Immunoelectron microscopic analysis demonstrated no statistically significant difference in the expression levels of κ and λ light chains (**Figure 1C**).

Based on the clinical presentation, laboratory findings, and renal pathology, the patient was diagnosed with: podocytopathy; nephrotic syndrome; acute kidney injury and at the same time suspected but unconfirmed B-cell lymphoproliferative disorder was also be considered. A multidisciplinary approach was adopted. Supportive care included a low-salt diet and fluid restriction. Immunosuppressive therapy with the CD20 monoclonal antibody Obinutuzumab (a total dose of 2g, given in two divided doses two weeks apart, each on June 7, 2024, and June 22, 2024). The patient was concurrently prescribed anticoagulant therapy and lipid-lowering agents. Given that his blood pressure was normal and his renal function was unstable, we did not administer ACEI/ARB medications.

The patients attended a follow-up visit at our outpatient clinic in the first month after receiving the second dose of Obinutuzumab. Within one month, he achieved complete remission of proteinuria, with urine protein excretion decreasing from 12.4 g/d to 0.06 g/d. Serum albumin levels increased from 23.9 g/L to37.7 g/L, and serum creatinine normalized from 180 μmol/L to 75 μmol/L (eGFR:84.7mL/min/1.73m2). The immunoglobulin levels showed a significant elevation of IgG, while IgM decreased to near the upper limit of the normal range(**Table 2**). Although persistent IgM-κ M protein was noted on serum and urine immunofixation electrophoresis. Unfortunately, as a farmer with limited financial resources, due to the absence of significant discomfort, the patient declined to adhere to regular hospital follow-up visits.

**Table 2:** Key examination indices during patient monitoring.

Index/time	2024/5/15-18 (First visit)	2024/6/5 (Prior to the first OBI Adm.)	2024/6/20 (Prior to the second OBI Adm.)	2024/7/23 (OPD follow-up)
24 UP (g/d) (Ref: 0-0.15g/d)	12.4	13.2	16.44	0.06
Alb (g/L) (Ref: 40-45g/L)	23.9	17.6	12.4	37.7
Cr (umol/L) (Ref: 57-111umol/L)	180	162	151	75
IgG (g/L) (Ref: 8, 16g/L)	< 2.5	< 2.5	< 2.5	7.82
IgA (g/L) (Ref: 0.7, 3.3g/L)	2.68	2.71	2.54	2.31
IgM (g/L) (Ref: 0.5, 2.2g/L)	3.33	3.51	3	2.31

OBI, binutuzumab; IV, intravenous; Adm, Administration; OPD, outpatient department; 24h UP, 24 hour urinary protein; Alb, Albumin; Cr, Creatinine; IgG/IgA/IgM, Immunoglobulin G/A/M; Ref, Reference range.

At one-year telephone follow-up, the patient reported complete resolution of lower extremity swelling and return to normal daily activities, stating he felt “good.” A year post-treatment (May 2025), the patient underwent a basic physical examination at a local hospital, which revealed normal results for urine tests, blood routines, and liver and renal function. Abdominal ultrasound was normal. These findings further confirmed the efficacy of the current treatment regimen and the stability of the disease. He remains aware of the need for long-term monitoring and would return if symptoms recur.

## Discussion

This case showed the clinical challenges encountered when dealing with patients presenting complex multi, system manifestations. This elderly patient was initially admitted due to intermittent bilateral lower limb edema, and preliminary diagnosed as nephrotic syndrome. He had no history of hypertension, diabetes mellitus, hepatitis, or autoimmune diseases, and no abnormalities in these aspects were detected. Therefore, nephrotic syndrome secondary to the aforementioned conditions was not considered. The emergence of an IgM lambda monoclonal component in both the patient’s blood and urine, with a slightly excess blood IgM broadened the differential diagnosis. Subsequent bone marrow examination did not support the diagnosis of these hematologic diseases, yet revealed the presence of malignant lymphoma cells within the bone marrow. However, these malignant lymphoma cells did not exhibit the phenotypic characteristics typical of any specific lymphoma. In the absence of peripheral lymphadenopathy or other clinical symptoms, the patient was diagnosed with suspected but unconfirmed B-cell lymphoproliferative disorder rather than overt lymphoma. The presence of monoclonal protein primarily raised the differential diagnosis of monoclonal gammopathy of renal significance (MGRS). Renal pathology is crucial for this differential diagnosis. While electron microscopy showed rare mesangial deposits, both immunofluorescence and immunoelectron microscopy failed to demonstrate light chain restriction (Kappa/Lambda), and there were no findings of complement activation or thrombotic microangiopathy. These pathological features do not support classical MGRS. This aligns with previous observations that not all patients with detectable M proteins actually develop MGRS. *Zi-hao Yong* et al. conducted an analysis of the kidney histopathologic spectrum associated with MGRS. Their study focused on the patients presented with monoclonal gammopathy (demonstrated by a monoclonal spike on serum and/or urine immunofixation tests) and underwent kidney biopsy over a 20-year period at a single hospital. Ultimately, they found that only 38% of these patients had MGRS lesions, while 62% had non-MGRS kidney diseases (7). Based on this premise, another differential diagnosis also existed for this particular patient: the M-proteinmia and kidney disease may existed independently of each other, which suggested the potential existence of monoclonal gammopathy of undetermined significance (MGUS) along with podocytopathy.

The detection of malignant B-cell clones in bone marrow also raised the possibility of a potential paraneoplastic podocytopathy. Although HE staining of the patient’s renal pathology revealed no atypical lymphocytes, offering no evidence of renal infiltration by lymphomatous cells, the suspected but unconfirmed B-cell lymphoproliferative disorder could induced aberrant cytokine profiles and immune dysregulation, which may also contribute to the development of podocytopathy (8). In the previous research, podocytopathies, particularly MCD, are well-documented in lymphoma patients, especially Hodgkin’s lymphoma where proposed mechanisms include T-cell dysfunction and c-mip dysregulation (1, 9, 10). Similar associations with NHL subtypes have led to proposals that podocytopathy may serve as a paraneoplastic glomerulonephritis (11–13). This hypothesis is supported by reports of nephrotic syndrome remission following lymphoma-directed chemotherapy (including Fludarabine, Cyclophosphamide, and Rituximab(FCR) therapy, Rituximab, Cyclophosphamide, Adriamycin, Vincristine, and Prednisone (R-CHOP) therapy et al.) (4, 9, 11, 14–20).However, applying this framework to our case faces critical limitations. First, the patient has suspected but unconfirmed B-cell lymphoproliferative disorder, not a confirmed lymphoma, the pathogenic potential of an indolent subclinical clone remains speculative. Second, the therapy itself confounds interpretation: Obinutuzumab is not only a lymphoma therapy but also an established treatment for primary podocytopathy. Finally, coincidence remains possible indolent B-cell clones and podocytopathy may occur independently in this elder patient. The intrinsic connection and corresponding therapeutic strategy between podocytopathy and suspected but unconfirmed B-cell lymphoproliferative disorder requires in-depth investigation, which is of great significance for this group of patients.

Such a diagnostic approach holds particular significance in formulating an effective therapeutic plan for our patient. Treatment was imperative for his podocytopathy. Under light microscopy, no glomeruli with segmental sclerosis were found in the patient’s renal tissue. Electron microscopy showed diffuse fusion of podocyte foot processes along with microvillous transformation, which aligned more closely with the manifestations of MCD. However, due to the relatively large number of sclerotic glomeruli (7/24) in this patient (though these sclerotic glomeruli may be age-related), FSGS could not be entirely excluded. For an isolated podocytopathy, options include corticosteroids, calcineurin inhibitors (CNI), and CD20 monoclonal antibodies (21). Given the patient’s advanced age, long-term, high-dose corticosteroid would increase the risks of infection, hyperglycemia, and osteoporosis. CNI were also unsuitable due to acute kidney injury at presentation. Considering the patient’s suspected but unconfirmed B-cell lymphoproliferative disorder, we sought a treatment strategy that could offer a ‘dual benefit’ by addressing both the renal and the underlying clonal B-cell disorder. We therefore selected the CD20 monoclonal antibody Obinutuzumab for this patient. In fact, CD20 monoclonal antibodies have emerged as a therapeutic option in podocytopathy, with studies supporting their use in both initial treatment and refractory MCD (22–24). The therapeutic effect of CD20 monoclonal antibodies in podocytopathy may be associated with limiting the secretion of pathogenic cytokines to alleviate podocyte injury, and may involving the reduction of autoantibody production, such as decreasing anti-nephrin antibodies in MCD (23, 24). While Rituximab, the first-generation anti-CD20 antibody, has been used in podocytopathy with demonstrated efficacy in inducing remission and reducing steroid exposure, Obinutuzumab offers distinct advantages as a next-generation therapy (22). Obinutuzumab is a humanized, glycoengineered type II anti-CD20 monoclonal antibody that targets a different epitope on CD20 than Rituximab, enabling direct cell death and enhanced antibody-dependent cell-mediated cytotoxicity. *In vitro* studies confirm its greater B-cell depleting potency (25). The phase III GALLIUM study showed improved progression-free survival with Obinutuzumab versus Rituximab in follicular lymphoma, attributed to its enhanced B-cell cytotoxicity (26).In addition, clinical studies demonstrate Obinutuzumab therapy lead to a rapid complete remission in MCD patients (23) and is effective in patients with membranous nephropathy or MCD who are refractory to Rituximab (23, 24). Thus, Obinutuzumab was selected as the optimal therapeutic choice to simultaneously address the podocytopathy and the suspected underlying clonal disorder. The subsequent clinical outcome was striking, and the rapidity of this response warrants discussion of the underlying mechanisms. The patient achieved complete remission of proteinuria within one month, with renal function returning to normal levels. One-year telephone follow-up and subsequent clinical examinations confirmed sustained renal remission. Moreover, he did not experience any adverse reactions that might be related to Obinutuzumab. The therapeutic response may reflect a direct effect of Obinutuzumab on podocytopathy, indirect elimination of pathogenic clonal B-cells (supported by declining IgM levels), or a synergy of both mechanisms. In this specific case, we cannot definitively distinguish between these possibilities. Nevertheless, the excellent clinical outcome underscores the potential utility of Obinutuzumab in similar complex cases.

Although this case demonstrates a successful application of Obinutuzumab in treating and controlling both podocytopathy and suspected but unconfirmed B-cell lymphoproliferative disorder, there were still some limitations. First, the diagnosis of his suspected but unconfirmed B-cell lymphoproliferative disorder lacks precision. It was based on the malignant lymphoma cells in his bone marrow biopsy. Since the patient’s refusal, no gene tests with discriminatory significance were conducted, particularly those targeting the elevated IgM levels, such as tests for genotypes including MYD88and L265P. Second, owing to the fact that the patient resided in a remote and economically underdeveloped area, he was unable to undergo detailed follow-up. This patient needs to undergo more in-depth follow-up visits for assessment.

## Conclusion

This is the first report of Obinutuzumab successfully treating podocytopathy with concurrent suspected but unconfirmed B-cell lymphoproliferative disorder. The patient achieved complete and sustained renal remission within one month. While the underlying mechanism, whether direct immunomodulation, B-cell clone control, or both, remains uncertain. The excellent outcome supports Obinutuzumab as a promising therapeutic option for such complex cases. Further research into the link between clonal B-cell disorders and podocytopathy is needed.

## Data Availability

The original contributions presented in the study are included in the article/supplementary material. Further inquiries can be directed to the corresponding author.
